# DNA- and RNA- Derived Fungal Communities in Subsurface Aquifers Only Partly Overlap but React Similarly to Environmental Factors

**DOI:** 10.3390/microorganisms7090341

**Published:** 2019-09-11

**Authors:** Ali Nawaz, Witoon Purahong, Martina Herrmann, Kirsten Küsel, François Buscot, Tesfaye Wubet

**Affiliations:** 1 Helmholtz Centre for Environmental Research–UFZ, Department of Soil Ecology, 06120 Halle (Saale), Germany; witoon.purahong@ufz.de (W.P.); francois.buscot@ufz.de (F.B.); 2 Helmholtz Centre for Environmental Research–UFZ, Department of Community Ecology, 06120 Halle (Saale), Germany; 3 Institute of Biology, University of Leipzig, 04103 Leipzig, Germany; 4 Institute of Biodiversity, Friedrich Schiller University Jena, Dornburger Straße 159, 07743 Jena, Germany; martina.herrmann@uni-jena.de (M.H.); kirsten.kuesel@uni-jena.de (K.K.); 5 German Centre for Integrative Biodiversity Research (iDiv) Halle-Jena-Leipzig, Deutscher Platz 5e, 04103 Leipzig, Germany

**Keywords:** total fungi, active fungi, aquatic fungi, aquifers, ITS, illumina sequencing, subsurface biosphere

## Abstract

Recent advances in high-throughput sequencing (HTS) technologies have revolutionized our understanding of microbial diversity and composition in relation to their environment. HTS-based characterization of metabolically active (RNA-derived) and total (DNA-derived) fungal communities in different terrestrial habitats has revealed profound differences in both richness and community compositions. However, such DNA- and RNA-based HTS comparisons are widely missing for fungal communities of groundwater aquifers in the terrestrial biogeosphere. Therefore, in this study, we extracted DNA and RNA from groundwater samples of two pristine aquifers in the Hainich CZE and employed paired-end Illumina sequencing of the fungal nuclear ribosomal internal transcribed spacer 2 (ITS2) region to comprehensively test difference/similarities in the “total” and “active” fungal communities. We found no significant differences in the species richness between the DNA- and RNA-derived fungal communities, but the relative abundances of various fungal operational taxonomic units (OTUs) appeared to differ. We also found the same set of environmental parameters to shape the “total” and “active” fungal communities in the targeted aquifers. Furthermore, our comparison also underlined that about 30%–40% of the fungal OTUs were only detected in RNA-derived communities. This implies that the active fungal communities analyzed by HTS methods in the subsurface aquifers are actually not a subset of supposedly total fungal communities. In general, our study highlights the importance of differentiating the potential (DNA-derived) and expressed (RNA-derived) members of the fungal communities in aquatic ecosystems.

## 1. Introduction

With a global diversity around 2.2–3.8 million species [1], fungi represent an important group of microbial eukaryotes. They play significant ecological roles in environmental processes and ecosystem services as decomposers, pathogens, and symbionts. However, out of this huge estimated diversity, fewer than 10% (147,076) of the fungal species are formally described [2]. Such disparity is primarily attributed to the limitation of culture-dependent methods for diversity studies, but also to the fact that many fungal habitats are still unexplored, in which “missing fungi” could be found [3].

Since the advent of culture-independent molecular methods and next-generation sequencing (NGS) technologies (which typically do not require cultivation), microbial ecologists have deepened their studies and extended them to previously unexplored habitats. This in particular helped in broadening the scope of aquatic mycology [4]. One of those unexplored fungal habitats are pristine aquifer systems within the terrestrial subsurface, which provide clean drinking water to more than 25% of the world population [5,6]. However, despite the immense significance of these aquifers to mankind and the vital ecological roles fungi play in aquatic ecosystems, fungal specific studies applying culture-independent high-throughput sequencing (HTS) approaches in natural subsurface aquatic environments are still rare [7].

Importantly, most available studies on fungal communities in groundwater aquifers or related aquatic environments have used environmental DNA (eDNA) [8,9,10]. However, targeting eDNA does not only give a clue to viable cells (living and dormant) but also to resting spores, dead hyphae, or even relic DNA, and consequently the richness of different taxa in the available datasets are inflated [11,12] which can lead to inaccurate interpretation of the sequencing data in the ecological context. In contrast, by extracting transcribed RNA and reverse transcriptase polymerase chain reaction (RT-PCR) based sequencing, the active members of fungal communities can be targeted [4,13,14]. This approach can even be used for targeting the universal DNA barcode region for fungi, the nuclear ribosomal internal transcribed spacer region (ITS) [15], and the few available studies showed that rRNA derived fungal communities differ from those derived from rDNA at the levels of richness, abundance of certain taxa and overall community composition [14,16,17,18,19]. However, such studies comparing total and active fungal communities have mostly been carried out either in surface soils from different biomes [14,16,17,20], or in communities associated with different plant species [19,21], but equivalent studies in subsurface aquatic ecosystems are largely missing.

Therefore, in the present study, we aimed to address this fundamental knowledge gap. For this, we collected groundwater samples from the two pristine aquifers assemblages of the Hainich Critical Zone Exploratory (Hainich CZE, Thuringia, Germany) [22] and applied high-throughput paired-end Illumina sequencing of the fungal nuclear ribosomal internal transcribed spacer 2 (ITS2) region to the DNA and RNA extracted from water samples. Current knowledge from other ecosystems has documented the differences between “total” and “active” fungal communities and it has been proposed that in any given environment only a part of the fungal community is metabolically active [4]. Accordingly, we hypothesized that in terrestrial subsurface aquifers, RNA-derived fungal communities (active) will be different from the DNA-derived communities (total) and that the active fungal community will be a subset of total fungal community. We tested these hypotheses by comparing (i) the species richness in the DNA- and RNA-derived fungal communities, (ii) the taxonomic composition of the DNA- and RNA-derived communities, (iii) the corresponding influence of sets of environmental factors known to shape fungal communities, and (iv) the respective distributions of fungal functional groups in the total and active communities.

## 2. Materials and Methods

### 2.1. Study Site

The study was conducted in Hainich Critical Zone Exploratory (Hainich CZE) within the framework of the Collaborative Research Centre AquaDiva (CRC 1076 AquaDiva). The geographical and geological characteristics of the study site are comprehensively described in Küsel, et al. [22]. Briefly, as a central feature of the Hainich CZE, a groundwater transect was built in the Hainich National Park in the federal state of Thuringia, Germany. The ~6 km-long groundwater transect (gently inclined, 2 degrees) accesses the subsurface habitats at several relief positions or depths, and covers three different land-use types, i.e., forest, grassland, and cropland. A graphical illustration of the study site (Hainich CZE) and onsite photos of the groundwater sampling wells (H3, H4 and H5) is provided in Figure S1.

The multi-storey subsurface aquifers system is used regionally as a groundwater resource and its recharge areas are located in different land use types [22,23]. This multi-storey aquifer system can be categorized into two major aquifer assemblages, the lower aquifer assemblage (HTL), and the upper aquifer assemblage (HTU), hosting nine aquifer storeys [23]. The basic hydro-chemical characteristics of the sampling sites in the lower and upper aquifer assemblages are described in Nawaz, et al. [24].

### 2.2. Groundwater Sample Collection

During regular four-weekly joint sampling campaigns in December 2014, March 2015 and June 2015, we collected 20 groundwater samples for physio-chemical and biological analysis. Depending on the sufficient water levels in the monitoring wells and the field accessibility during joint sampling campaigns, water samples from the lower and upper aquifer assemblage were collected from seven permanently water-bearing monitoring wells at sites H3, H4, and H5. Prior to sample collection, the groundwater was pumped out and discarded, using a submersible sampling pump (MP1, Grundfos, Denmark) until the physico-chemical parameters stabilized.

For molecular analysis (DNA and RNA extraction), the collected groundwater was thoroughly mixed, transferred into sterile glass bottles, stored at 4 °C, and transported to the laboratory within one hour. For DNA extraction, 5000–6000 mL and for RNA extraction approximately 2000–3000 mL of the homogenized groundwater were filtered through 0.2 µm polyethersulfone (Supor, Pall Corporation, Bad Kreuznach, Germany) and polycarbonate filters (Nuclepore, Whatman; Merck, USA) respectively. The filters were then transferred into sterile reaction tubes and stored at −80 °C until subsequent nucleic acid extraction.

### 2.3. Physico-Chemical Analysis of Groundwater Samples

For all the water samples, physico-chemical parameters, i.e., extraction temperature, pH, dissolved oxygen, redox potential, and specific electrical conductivity were measured on site in a flow-through cell using respective probes as explained in Küsel, et al. [22]. In situ groundwater temperatures were obtained by permanently installed data-loggers (see Kohlhepp et al. 2017). The concentrations of total organic carbon (TOC), nitrate, nitrite, ammonium, and sulfate ions were measured as described in Nawaz, et al. [24].

### 2.4. Nucleic Acid Extraction, Amplicon Library Preparation and Pair-End Illumina Sequencing

Extraction of genomic DNA and total RNA from the filter disks was carried out by using the PowerSoil DNA Isolation Kit and PowerWater RNA Isolation Kit (MO BIO Laboratories Inc., USA), following the manufacturer’s protocol. In addition to on-column DNase treatment included in the protocol of the PowerWater RNA Isolation kit, RNA extracts were treated with TurboDNA free (Thermo Fisher Scientific, USA) and checked for PCR amplification using universal fungal primers to rule out the presence of any traces of DNA. The RNA extracts were then transcribed into cDNA using the NEBNext first strand synthesis kit (New England BioLabs, USA) following the manufacturer’s protocol.

To target total (DNA) and active (RNA-cDNA) fungal communities in the collected groundwater samples, an ITS2 library was constructed using the primer combination fITS7 [25] and ITS4 [26], which anneal to the 5.8S and LSU rRNA genes, respectively. The PCR reaction mix, library preparation, and the PCR amplification were performed as previously explained in Nawaz, et al. [24]. The paired-end Illumina MiSeq Sequencing was done at LGC Genomics Berlin by using V3 Chemistry (Illumina).

### 2.5. Bioinformatics Processing of Sequence Data

The raw reads generated by the Illumina MiSeq sequencing platform were processed using MOTHUR (v.1.39.5) [27] and OBI Tools (v.1.1.20) [28] software suits as previously explained in Nawaz, et al. [24]. The sequencing data derived from RNA samples (representing the active communities and is available under Bioproject number PRJNA436133) was analyzed together with the newly generated sequencing data derived from the corresponding DNA samples. Briefly, forward and reverse raw reads from the same sample were assembled by using the simple-Bayesian algorithm with a minimum overlap of 20 nucleotides as implemented in PANDAseq (v.2.8.1) [29]. Chimeric sequences were removed using the UCHIME algorithm [30] as implemented in MOTHUR. The resulting reads were then clustered into operational taxonomic units (OTUs) [31] using the CD-HIT-EST (v.4.7) algorithm [32] at a threshold of 97% sequence similarity. The OTU representative sequences were taxonomically assigned against the reference sequences from the UNITE database (version v.7.0) [33] using the naïve Bayesian classifier [34] as implemented in MOTHUR using the default parameters.

To have an additional quality control step, fungal reads were further quality filtered using ITSx (v. 1.0.11) [35] to remove 5.8S and 28S fragments and any non-fungal reads from the dataset, which improved downstream fungal analyses. The taxonomic assignments of the fungal OTUs based on UNITE v.7.0 is provided in Table S1. The fungal ITS2 raw sequence dataset is deposited in the National Center for Biotechnology Information (NCBI) Sequence Read Archive (SRA) and is available as bioproject number PRJNA436133 (RNA-derived dataset) [24] and PRJNA560486 (DNA-derived dataset).

### 2.6. Statistical Processing

The R [36] and PAST (v2.17) software [37] were used for the data analysis. The fungal sequences were rarefied to common sequence depths per sample to minimize the effects of the varying number of fungal sequences while comparing the DNA- and RNA-derived communities. A paired sample t-test (implemented in PAST v2.17) was used to compare the means of fungal-observed richness and estimated richness between the DNA- and RNA-derived communities. Sample-based individual rarefaction curves [38] were generated for all the DNA- and RNA- based samples to assess the sampling efforts by using the function “diversity” in PAST. Prior to the beta diversity analysis, the rare OTUs (singletons, doubletons, and tripletons) were removed from the total OTU matrix to avoid any potential PCR artifacts and sequencing errors. In order to assess the effect of removing the rare OTUs, we performed a Mantel test using Jaccard similarity measures with 999 permutations to assess the correlations between the whole matrix and a matrix excluding the rare OTUs [8]. The result indicated that the removal of rare OTUs from the total community did not affect the fungal community composition (R_Mantel_ = 0.999, *p* = 0.001). Therefore, the final dataset without rare OTUs was used for further statistical analysis unless otherwise stated.

To minimize the effect of differences in the abundance measure of different OTUs in the data set, we used presence/absence data for all the community composition analysis. To cluster the specific and shared fungal OTUs into a graphical illustration, Gephi software tool was used [39]. To visualize the fungal community composition, we used two-dimensional NMDS (non-metric multidimensional scaling) ordination based on the Jaccard dissimilarity index using the vegan package [40]. To test the significance of the differences between DNA-and RNA-derived fungal communities, we performed non-parametric multivariate analysis of variances (NPMANOVA) using PAST with a Bonferroni-corrected *p* value. Proportional Venn diagrams were calculated using BioVenn [41]. For all the analysis, a *p*-value of <0.05 was considered as significant.

### 2.7. Functional Analysis

The representative sequences of the dominant fungal OTUs detected in this study were used to assign the functional or ecological groups using the FUNGuild database (v. 1.1) [42].

## 3. Results

### 3.1. Illumina Sequencing of DNA- and RNA-Derived Fungal Communities

Paired-end Illumina sequencing of 40 fungal ITS2 libraries from 20 groundwater samples representing total and active fungal communities, produced a total of 2,299,575 paired-end reads with correct primer sequences. Sequential bioinformatics filtering of low-quality, short length, and chimeric sequences resulted in a total of 1,988,286 (86.46%) high-quality fungal ITS2 reads. The total number of reads varied between samples. Therefore, the dataset was rarefied to the smallest number of reads per sample to achieve a similar sequence depth of 1024 reads. After clustering the reads at 97% sequence similarities and removal of non-target reads, the DNA-derived community was represented by a total of 20,341 fungal reads, while the RNA-derived community was represented by 19,490 reads. After strict quality control pipeline filters during initial bioinformatics analysis and subsequent ITSx filtering of the fungal reads, we obtained a total of 14,421 reads from the DNA based samples and 8889 reads from corresponding RNA based samples. Sample-based rarefaction curves of the number of OTUs at 97% sequence similarity vs the sequencing efforts (number of reads) from all the samples indicated that we have done exhaustive sequencing of the DNA-derived fungal communities. However, the sampling efforts curves for some of the RNA based samples did not seem to reach a plateau, which indicated an undiscovered community in those samples. The accumulation curves are provided in Figure S2 in supplementary materials.

### 3.2. Fungal Species Richness of DNA- and RNA-Derived Communities

**Figure 1:**
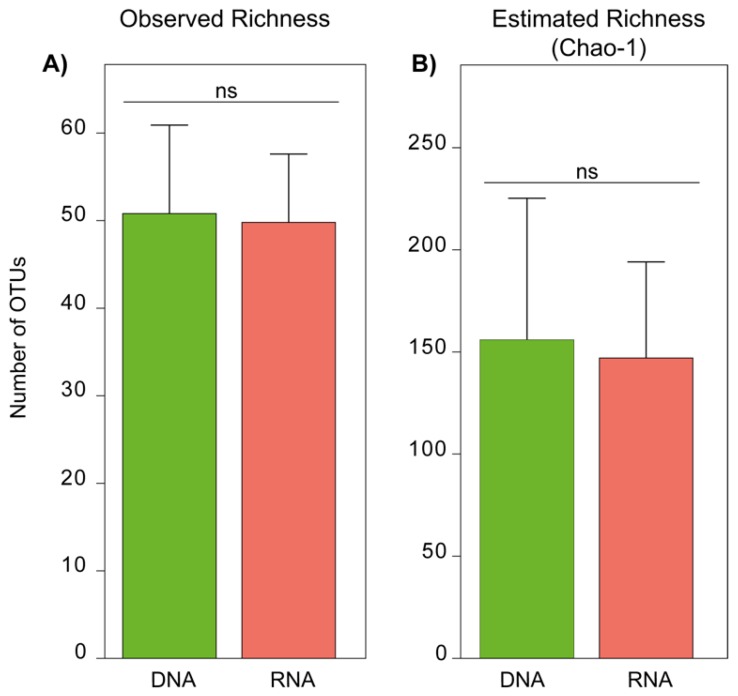
Observed richness and estimated richness for aquatic fungi in the DNA- and RNA-derived communities in subsurface aquatic habitat. (**A**) mean operational taxonomic unit (OTU) richness (number of OTUs detected) and (**B**) mean estimated richness (Chao-1) (mean + standard error (S.E.), *n* = 20). Significance in differences between mean values of observed richness and estimated richness (Chao-1) is based on paired t-test (*p* > 0.05).

The bar-graphs in Figure 1 shows the observed and estimated species richness of fungi in the DNA- and RNA-derived communities and subsequent statistical testing indicated that both observed and estimated fungal species richness was not significantly different between two fungal communities (paired t-test P_Observed_ = 0.896 and paired t-test P_Chao-1_ = 0.885).

**Figure 2:**
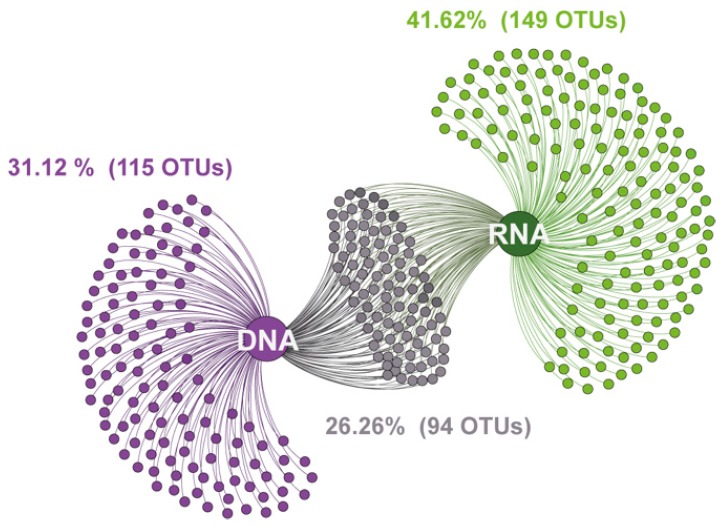
A graphical illustration showing 358 fungal OTUs detected in DNA- and RNA-derived communities from subsurface aquatic habitat. Distinct clusters show fungal OTUs exclusively present in DNA (purple) and RNA (green) derived communities and the OTUs shared (grey) between the two communities.

After removal of rare taxa (singletons, doubletons, and tripletons) from the dataset, we detected a total of 358 fungal OTUs from both DNA- and RNA-derived samples. However, the distribution of fungal OTUs was very different between the two communities detected by the DNA-derived and RNA-derived analyses, respectively. This difference is evident in Figure 2, which shows that only 26.26% (94) of fungal OTUs were shared between the DNA- and RNA-derived communities. Thereby, the percentage of the fungal OTUs present exclusively in the DNA-derived community was only 31.12% (115 OTUs), while 41.62% (149 OTUs) of the overall fungal community were exclusively observed in the RNA-derived communities.

### 3.3. Taxonomic Differences between DNA- and RNA-Derived Fungal Communities

**Figure 3:**
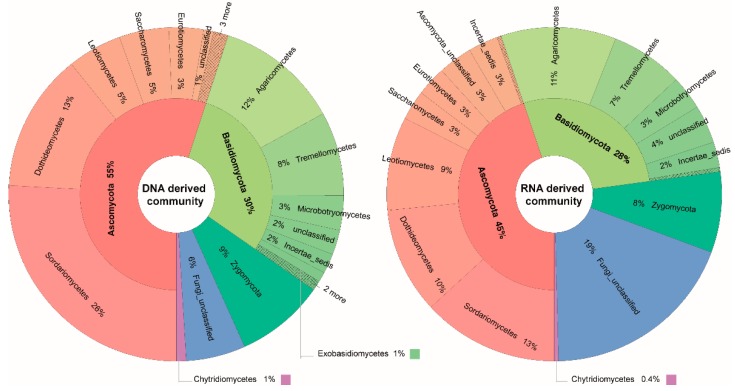
Krona charts illustrating the taxonomic distribution of fungal OTUs at phylum and class level, detected in the DNA- and RNA-derived communities from a subsurface aquatic habitat.

Taxonomic identification of the retrieved mycobiome from DNA- and RNA-derived communities of the subsurface aquifers revealed that the fungal OTUs mainly belonged to the Dikarya (Ascomycota 51% and Basidiomycota 28%). Zygomycota was represented by only 6% of fungal OTUs and Chytridiomycota by <1%. Specifically, in the DNA-derived fungal community the members of the phylum Ascomycota (55%) were most frequently detected, followed by Basidiomycota (30%), Zygomycota (9%) and Chytridiomycota ( <1%). Similarly, in the RNA-derived fungal communities, the highest contribution in the taxonomic distribution was by the members of the Ascomycota (45%), followed by Basidiomycota (28%), Zygomycota (8%) and Chytridiomycota (0.4%) (Figure 3). This illustrated that at the phylum level, there were no observable differences in the taxonomic distributions of DNA- and RNA-derived fungal communities in the aquifers.

**Figure 4:**
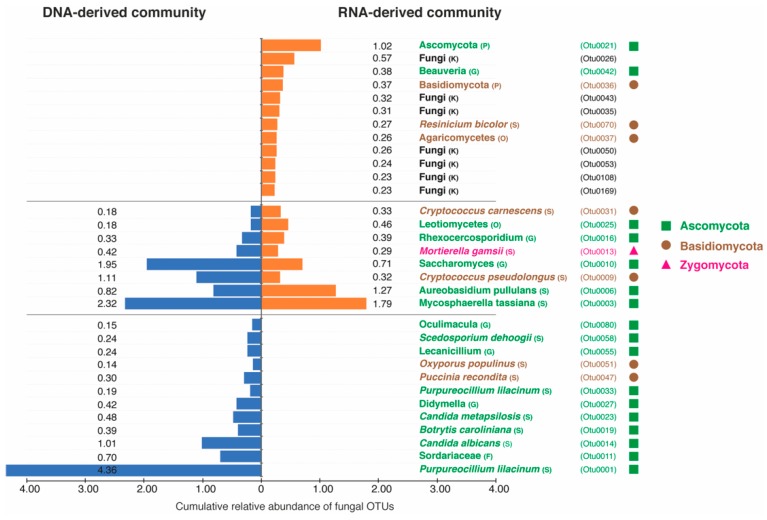
Histograms showing the top 20 fungal OTUs (based on cumulative relative abundance in 20 samples) and their cumulative relative abundance in the respective fungal communities derived from DNA and RNA. The OTUs are identified to the highest possible level of taxonomic resolution. The alphabets K, P, O, F, G and S are used to represent kingdom, phylum, order, family, genus, and species level of taxonomy. The orange and blue color bars represent the OTUs detected in the RNA- and DNA-derived communities respectively. The OTUs are color coded based on the legend provided on the right.

The taxonomic distributions of DNA- and RNA-derived fungal communities at the species level was further investigated based on the cumulative relative abundances of the top 20 frequently detected OTUs in the DNA- and RNA-based communities. We found that these OTUs exhibit distinct abundance levels between the two communities (Figure 4). Specifically, in the DNA-derived community, only 12/20 OTUs were specific to the DNA-derived community and 8 OTUs were shared with the RNA-derived communities. The OTUs exclusively present in the DNA-derived community mostly belonged to the phylum Ascomycota (10/12 OTUs) while only two OTUs belonged to phylum Basidiomycota. A similar distributional pattern of top abundant OTUs was observed in the RNA-derived community, where 12/20 OTUs were specific to the RNA-based community and 8 OTUs were shared with the DNA-derived community. However, in contrast to the DNA-derived community, the abundant OTUs unique to the RNA-derived community were almost equally represented by the phylum Ascomycota (2 OTUs) and Basidiomycota (3 OTUs). The remaining seven OTUs were only identified as fungi without further taxonomic assignment (Figure 4).

### 3.4. DNA- and RNA-Derived Fungal Communities and Significant Environmental Factors

We compared the overall fungal community composition between DNA- and RNA-derived communities and found that they were significantly different from each other (one-way NPMANOVA, F = 2.989, *p* = 0.001). Goodness-of-fit statistics from 3D-NMDS ordination provided meaningful insight into the most significant factors (*p* < 0.05) correlating with the DNA- and RNA-derived fungal community composition. This analysis indicated that NH_4_^+^, Ca^2+^ Cl^−^, and K^+^ were the environmental factors significantly correlating with the DNA-derived fungal community, while for the RNA-derived fungal community, the same factors plus Mg^2+^ were significantly correlated (Table 1).

### 3.5. Distinct Functional Guilds of DNA- and RNA-Derived Fungal Community

**Figure 5:**
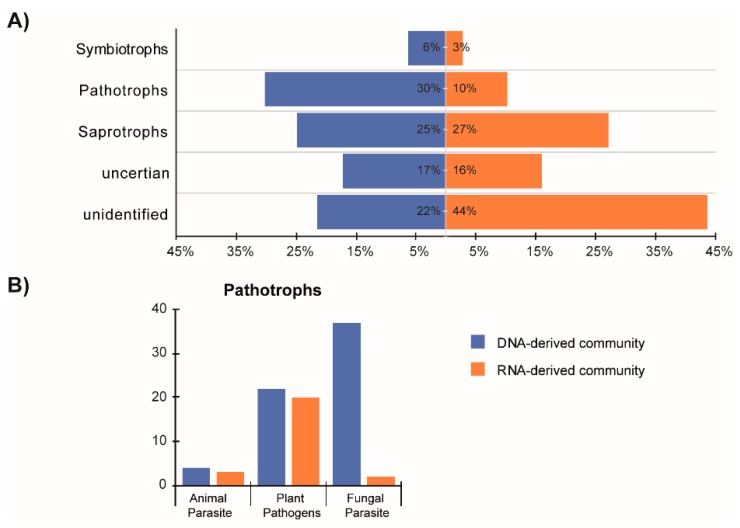
Histograms showing (**A**) the percent distribution of fungal functional guilds OTUs classified based on FUNGuild database in the DNA- and RNA-derived fungal communities. (**B**) Histograms representing the number of fungal OTUs belonging to specific pathogenic groups (i.e., animal parasite, plant pathogen and fungal parasite) within the pathotrophic functional guild in the DNA- and RNA-derived fungal communities.

We identified three broadly defined functional guilds within the DNA- and RNA-derived fungal communities: saprotrophs (81 OTUs), pathotrophs (76 OTUs), symbiotrophs (16 OTUs), and in addition also fungi with either uncertain or unidentified functions (185 OTUs). The percent distributions of the fungal functional groups differed between the DNA- and RNA-derived communities (Figure 5A). Specifically, in the case of pathotrophs, 30% of DNA-derived community and only 10% of RNA-derived community was represented by this group. Moreover, in comparison to the DNA-derived community, 44% of the RNA-derived fungal community was represented by fungal species with unknown functions. This indicates that we either detected new fungal taxa with unknown functions in the aquifers or that there is a lack of reference sequences in the database. We dissected the pathotrophic functional guild to get a deeper insight into this group and found that the number of OTUs representing animal and plant pathogens were not different between the DNA- and RNA-derived communities, while a significant difference was observed concerning fungal parasites (Figure 5B).

**Figure 6:**
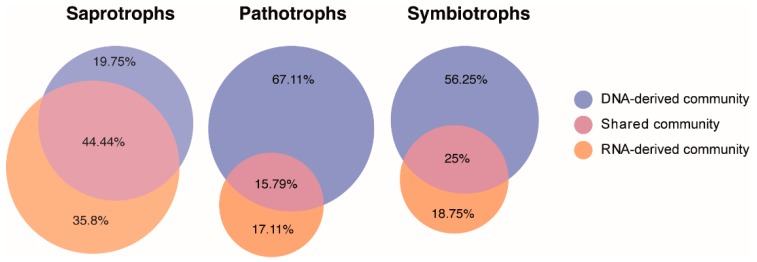
Proportional Venn diagrams presenting the distribution of shared fungal OTUs classified as saprotrophs, pathotrophs and symbiotrophs (based on FUNGuild classification) between DNA- and RNA-derived communities from a subsurface aquatic habitat.

Further deeper analysis of saprotrophs, pathotrophs, and symbiotrophs detected in the DNA- and RNA-derived fungal communities revealed that 35.8%, 17.11%, and 18.75% of the saprotrophs, pathotrophs, and symbiotrophs were exclusively detected in the RNA-derived community (Figure 6).

## 4. Discussion

Much of what is known about aquatic mycology has primarily been inferred from studies targeting eDNA. However, to link fungi and their activities to ecosystem processes precisely, it is important to disentangle metabolically active members (which are actually driving different ecological processes) from the total community. To our knowledge, by using metabarcoding based high-throughput sequencing approach, this is the first comprehensive comparative analysis of DNA- and RNA-derived fungal communities in groundwater aquifers in the terrestrial subsurface biosphere.

### 4.1. The Total and Active Fungal Communities Have Similar Structure but Different Community Composition

Firstly, comparing the alpha diversity (observed and estimated richness) of total and active fungal communities, we found no significant differences. A non-significant differentiation of alpha diversity between total and active communities indicates a similar structural organization of the fungal community. Such similar structural organization in the total and active bacterial communities during anaerobic digestion process was also reported by De Vrieze, et al. [43]. Generally, active members of the microbial communities are those that grow, adapt, and respond to the changes in the local inhabited environments. Therefore, the RNA-based analysis of microbial communities are known to provide a snapshot of the active members of communities at specific timepoints [11], and observing a similar structural organization of total and active communities in our datasets might also be suggesting a higher activity timepoint requiring a higher active fungal community diversity.

Secondly, we found clear differences between DNA- and RNA-derived fungal communities in terms of the proportional distribution of different fungal phyla. These results are well supported by a recent study by Wegner, et al. [44], who found profound differences in the taxonomic profiles of metagenomes and metatranscriptomes of groundwater samples from Hainich CZE. Moreover, a limited overlap of common OTUs (Figure 3) while comparing fungal OTUs exclusively present in the DNA and RNA communities is also consistent with a previous study in forest soils by Baldrian, et al. [14], who reported that 18% of the total detected fungal OTUs were exclusively present in the RNA-derived community while only 2% were exclusively found in the DNA-derived community. A similar pattern of abundant bacterial OTUs from rhizosphere soil analysis were also reported by Li, et al. [45].

Furthermore, a significant differentiation in the predicted fungal functional guilds between DNA and RNA profiles and in the top abundant OTUs was found. This accentuates the fact that although the overall fungal alpha diversity (which is a qualitative measure) of total and active fungal communities does not differ significantly, the community composition (quantitative measure) is significantly different between the two communities. Such differences in total and active microbial community compositions were also reported from studies in forest soils [14,16,17], root-associated microbial communities [19], marine samples [46] and in the anaerobic digestion processes [43,47]. De Vrieze, et al. [43] suggested that multiple microbial species are able to occupy the same niche with only a part of them being active at each time point. But, this small fraction of the active species may be least represented in the larger DNA pool of the community. This assumption was confirmed by analyzing the relative abundance of top 20 OTUs from the DNA- and RNA-derived communities, as we found that the 20 abundant OTUs in the RNA-derived community were either not detected in the DNA-derived community or their relative abundance was negligible. This strongly indicates that infrequent rare taxa in the DNA-derived communities may be key active players evidenced in the RNA-derived communities [14,45].

Lastly, the significance of environmental factors in shaping the fungal communities in a variety of aquatic habitats has been demonstrated by number of studies [48,49,50,51,52]. Our study expands the current scope of information by demonstrating that the same set of environmental paraments (NH_4_^+^, Ca^2+^ Cl^−^, and K^+^) shape the composition of both DNA- and RNA-derived fungal communities in the pristine aquifers in the terrestrial subsurface biogeosphere. However, it cannot be excluded that the observed differentiation in the community composition between the DNA- and RNA-derived communities might be associated with physico-chemical parameters which we did not measure. Additionally, besides the roles of individual physico-chemical parameters, the observed shifts in relative abundance of different fungal taxa in a community, could also be driven by complex inter- and intra-kingdom microbial interactions based on competition or cooperation for nutrients [53]. Therefore, we suggest further experimental and statistical validation to associate any individual physico-chemical factor which is potentially responsible for any differential abundance of certain taxa between the DNA- and RNA-derived fungal communities in the aquifers.

### 4.2. Does the DNA-Derived Fungal Community Represent the Total Fungal Community?

As a general assumption, it is widely believed that the datasets generated by DNA based HTS approach represent a complete pool of the targeted species at the time of sampling. Therefore, DNA-derived microbial communities are largely considered as “total communities”. This generalization is primarily based on the notion that in a microbial community pool, there must be a DNA present for each expressed RNA and it should be detected/amplified during PCR amplification step (which is the foremost and integral step in the amplicon based HTS approaches). However, owning the fact that PCR is an enzymatic reaction which follows the rules of stoichiometry, PCR amplification of microbial taxa in a complex heterogeneous community with varying abundance cannot mirror the total communities coherently. This implies that during a PCR amplification process for the DNA-based community, the amplified products will be dominated by the abundant taxa representing mainly the potential or non-active communities as compared to the less abundant but active taxa. Our results also indicate a similar case, as we found that numerous fungal OTUs were only detected in the RNA-derived communities. This highlights the need to move to a non PCR-based analyses i.e., shotgun sequencing. However, owning the fact that metagenome or metatranscriptome based shotgun sequencing is difficult to apply for large sets of samples [54], and that whole genome data for their exhaustive exploitation are still under construction [55,56], performing studies that couple DNA- and RNA-based amplicon analyses would be an optimal choice for previously unexplored habitats. Such coupled analyses will not only help to reveal new taxa but also give a better view on the contribution of the active community in the functioning of the ecosystem. In this regard the present work points out that in the RNA-derived communities, a higher proportion of OTUs not corresponding to known taxa was found and the distribution of functional group was different in comparison to those of DNA-based communities. Hence, based on our findings and reasons mentioned above, this study unambiguously confirms that the DNA-derived fungal community does not represent the total community pool and the RNA-derived fungal community is not a complete subset of DNA-derived fungal community.

## 5. Conclusions

This research clearly demonstrates the differentiation between the DNA- and RNA-derived fungal communities in the aquifers. Based on the results from this study, we can conclude that although DNA-based analyses of fungal communities in aquatic ecosystems might provide a broader overview of the community (by targeting DNA from both living and dead/dormant species), but for a meaningful ecological interpretations of community level diversity and species composition analyses, it is also important to consider RNA-based analyses to better differentiate between fungal OTUs originating from living fungi versus dead/dormant species.

## Figures and Tables

**Table 1:** Goodness-of-fit statistics (R^2^) for factors fitted to the three-dimensional non-metric multidimensional scaling (3D-NMDS) ordination of DNA- and RNA-derived fungal community composition based on presence/absence data and Jaccard distance measure.

Factors	DNA-derived Community		RNA-derived Community	
	R^2^	P	R^2^	P
Extraction °T	0.16	0.10	0.15	0.11
EC25	0.14	0.14	0.15	0.11
pH	0.02	0.86	0.03	0.82
ORP	0.11	0.24	0.10	0.28
O_2_	0.09	0.33	0.10	0.27
NH_4_^+^	0.24	0.01 *	0.26	0.01 *
PO_4_	0.04	0.68	0.04	0.72
TIC	0.03	0.81	0.01	0.91
NO_3_^−^	0.06	0.49	0.06	0.50
SO_4_^2−^	0.10	0.27	0.11	0.22
Cl^−^	0.22	0.03 *	0.24	0.02 *
Ca^2+^	0.18	0.05	0.20	0.04 *
Fe_t_	0.08	0.41	0.08	0.38
K^+^	0.21	0.03 *	0.23	0.03 *
Mg^2+^	0.19	0.06	0.21	0.03 *
Na^+^	0.16	0.10	0.18	0.07
S_t_	0.10	0.28	0.10	0.23

EC: Electric conductivity, ORP: Oxidation reduction potential, TIC: Total inorganic carbon.

## Supplementary Materials

Supplementary materials can be found at https://www.mdpi.com/2076-2607/7/9/341/s1.
