# Supplementary material for: DNA- and RNA- Derived Fungal Communities in Subsurface Aquifers Only Partly Overlap but React Similarly to Environmental Factors
Source: Microorganisms. 2019 Sep 11;7(9):341. doi: 10.3390/microorganisms7090341 (PMC6780912; doi:10.3390/microorganisms7090341)
Supplement: Supplementary file 1 [file microorganisms-07-00341-s001.zip › 15.3_Supplementary_materials_microorganisms-587586.docx]

**Supplementary Materials:**

**Figure S1**


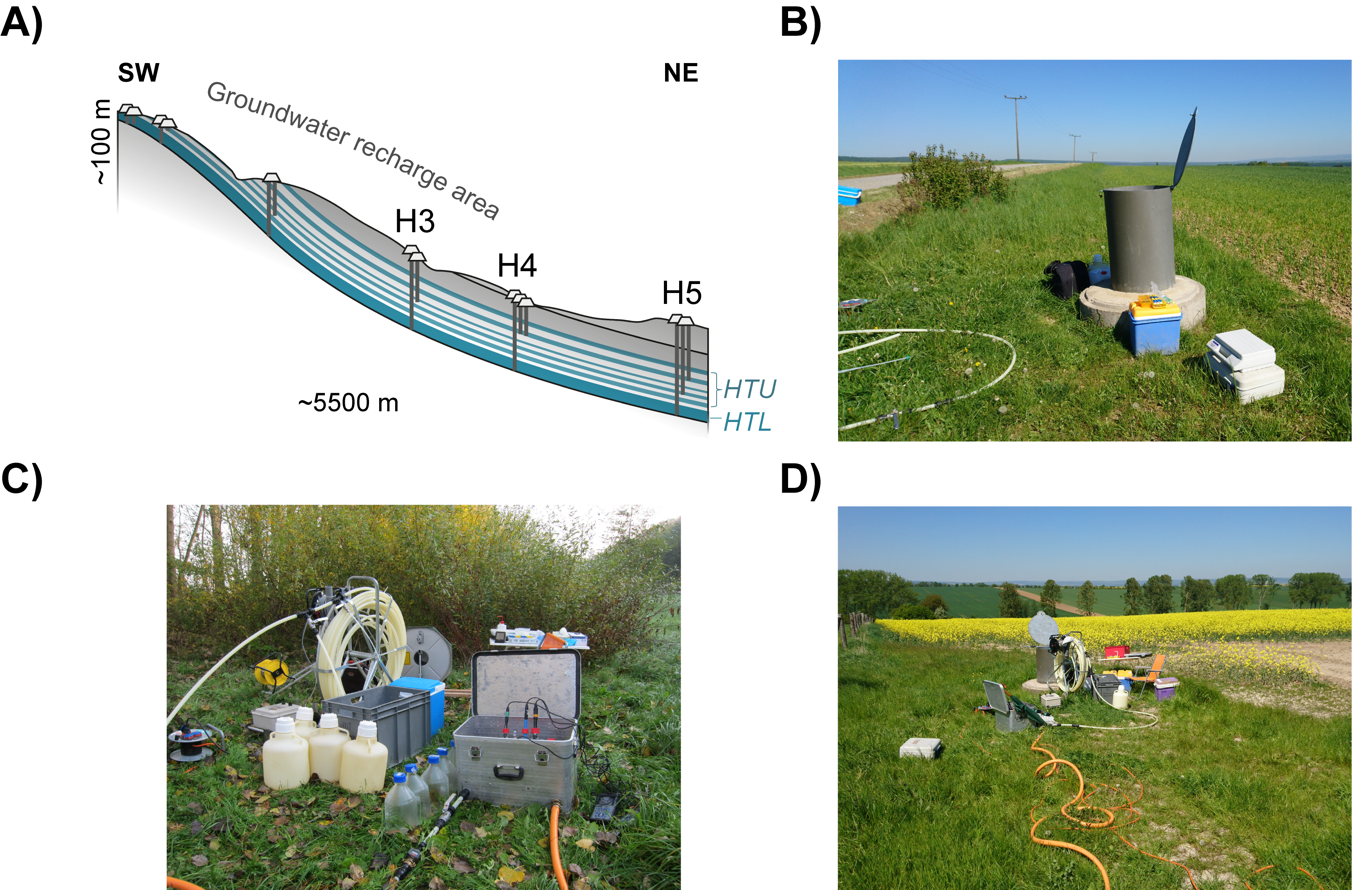


**Figure S1:** **(A)** Graphical illustration of the study site (Hainich CZE) Figure reproduced from Nawaz et al 2018. Water samples were collected from groundwater wells at site H3, H4 and H5. On site photos of groundwater monitoring well at site H3 **(B)**, H4 **(C)** and H5 **(D)**. Photos were taken by Falko Gutmann, Chair of Aquatic Geomicrobiology, Institute of Biodiversity Friedrich Schiller University Jena.

**Figure S2**


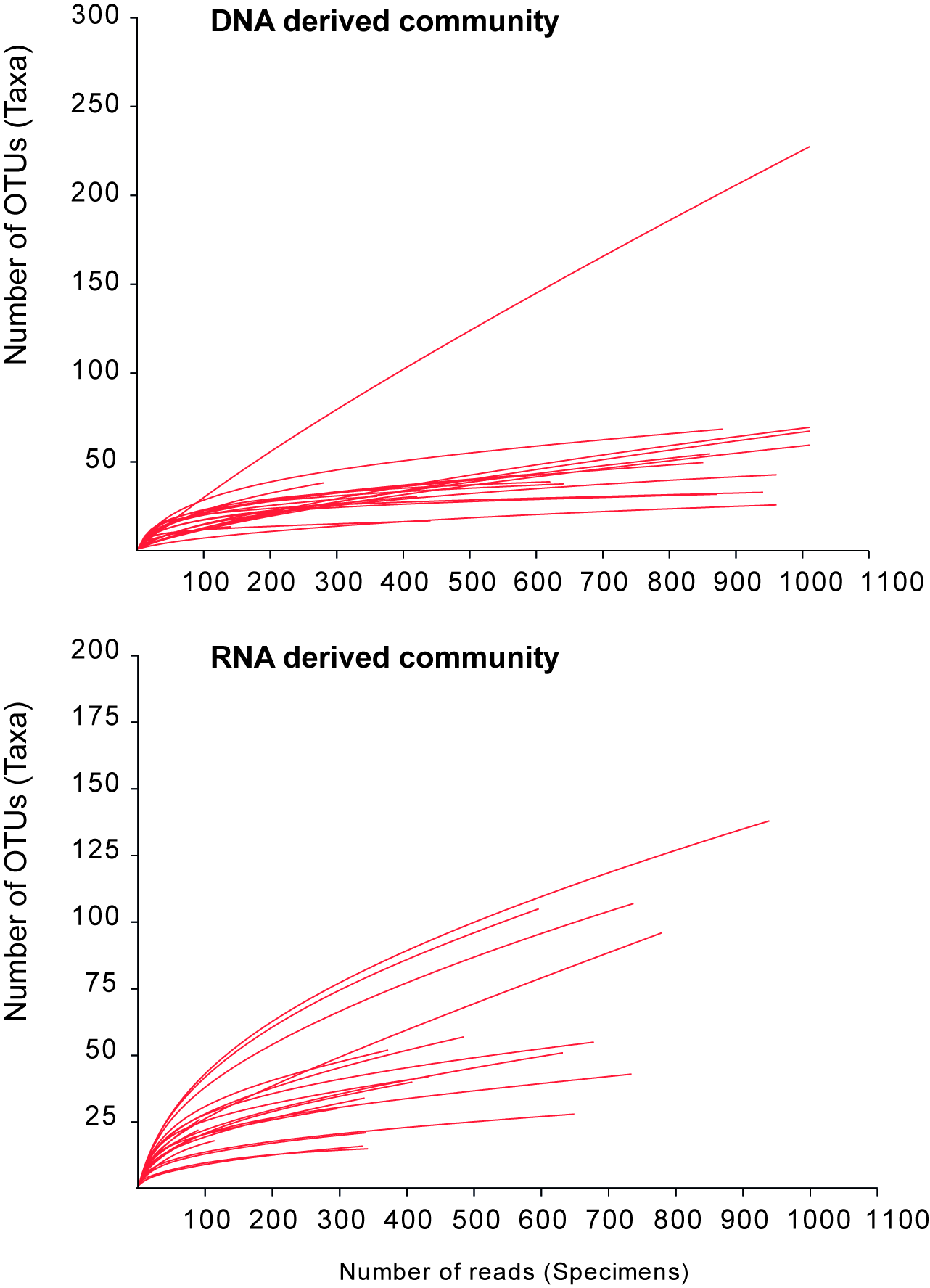


**Figure S2:** Sampling effort curves of DNA- and RNA-derived fungal communities from subsurface aquatic habitat showing the number of fungal OTUs (97% sequence similarity) as the function of number of reads analyzed.
